# Engineering Self-Assembled Nanomedicines Composed of Clinically Approved Medicines for Enhanced Tumor Nanotherapy

**DOI:** 10.3390/nano13182499

**Published:** 2023-09-05

**Authors:** Quzi Jiang, Luodan Yu, Yu Chen

**Affiliations:** 1 Shanghai Institute of Ceramics, Chinese Academy of Sciences, Shanghai 200050, China; jiangquziol@163.com; 2 University of Chinese Academy of Sciences, Beijing 100049, China; 3 School of Life Sciences, Shanghai University, Shanghai 200444, China; yuluodan@shu.edu.cn

**Keywords:** tumor chemotherapy, photothermal therapy, biosecurity, self-assembly, stimuli-responsive disassembly

## Abstract

The traditional nanocarriers are typically constructed to deliver anticancer agents for improving drug bioavailability and enhancing chemotherapeutic efficacy, but this strategy suffers from the critical issue of nanocarrier biosafety that hinders further clinical translation. In this work, a unique nanomedicine (PTX@ICG) has been rationally constructed by combining two clinically approved agents, i.e., paclitaxel (PTX) and indocyanine green (ICG), by a facile ultrasound-assisted self-assembly methodology. The formation of the nanostructure can effectively increase the enrichment of PTX and ICG molecules in the tumor site, and improve the utilization factor of hydrophobic PTX. Moreover, since the molecule interaction in PTX@ICG is mainly Van der Waals forces, the self-assembled structure can be spontaneously dissociated under laser irradiation and release PTX in situ to achieve safe tumor-targeted chemotherapy. Simultaneously, the released ICG can act as photothermic agents for photothermal therapy (PTT), thus combining chemotherapy and PTT to obtain an enhanced tumor nanotherapy via facile self-assembly. The synergistic chemo/photothermal tumor nanotherapy achieved the efficient tumor cell-killing effect and tumor-ablation ability, as systematically demonstrated both in vitro and in vivo. This work provides a distinct paradigm of the self-assembled nanomedicine design for effectively improving the drug bioavailability to achieve high antitumor efficacy.

## 1. Introduction

Due to the increasing incidence rate and the high hazards of cancer [1,2], it is a major medical problem that plagues humans [3,4]. Seriously, the dominant traditional treatments are problematic: surgery can be used to remove the lesions, but it easily leads to tumor recurrence and causes serious trauma to the body [5,6]; chemotherapy cannot achieve targeted therapy and usually causes toxic side effects on normal organs [7,8]; and radiotherapy can clear the pathological cell pointedly, but it will cause lesions in nearby tissues [9]. As a result, developing an efficient cancer-therapeutic modality that can realize targeted therapy, does not cause side effects, and can complete tumor eradication is highly necessary at the current stage. 

Recently, the composite nanosystems with nanocarriers and drugs [10,11,12,13,14] are focused on cancer treatment, which features the following merits. For instance, these nanocomposites can dissociate under external conditions, which guarantees the targeted therapy [15]. The versatility, size effect, and diverse morphological structures [16] of these nanocomposites make it possible for them to achieve the effective therapeutic function by a combination of multiple treatment methods [17,18] that show a prolonged body-circulation period and an enhanced permeability and retention effect (EPR effect) [19,20]. Therefore, this nanomedicine-mediated treatment potentiates its development and broad application in the field of cancer diagnosis and therapy.

To achieve the high performance and non-side effects of the composite nanosystems, it is imperative to construct biocompatible nanoscale carriers for loading small molecular chemotherapeutic drugs [21,22,23], photosensitizers [24,25,26], and other functional molecules [27,28]. The broad reports have shown the application potential of diverse nanosystems, including IrO_x_- [29], Fe_3_O_4_- [30,31], and MOF-based nanocarriers [32] loading with doxorubicin (DOX) [33], curcumin [34], and fluorouracil [35]. Nevertheless, due to the relatively preliminary research of these available nanomaterials and lack of data support for biochemical research and clinical applications, these nanocarriers can easily cause crucial issues on degradability and biocompatibility [36,37], which means that the biosafety of these nanocarriers, as applied in the human body, is difficult to guarantee at the current stage. Comprehensively, it is of high significance to develop a facile method to synthesize intelligent multifunctional nanomedicine which is composed of all clinical drugs approved by the Food and Drug Administration (FDA) without nanocarriers, thus ensuring its therapeutic effect, biosecurity, and biocompatibility [38].

In this work, we adopted the biosafe indocyanine green (ICG) with diagnostic and therapeutic functions and the highly effective tumor-therapeutic medicine paclitaxel (PTX) to construct a new type of nanomedicine (PTX@ICG) without nanocarriers through weak interaction force (hydrophilic-hydrophobic self-assembly) via an ultrasound-assisted synthesis method, which avoided the use of methanol and was easier to prepare the nanoparticles than the reported solvent evaporation method [38]. Due to the EPR effect, the designed PTX@ICG can be enriched in the tumor tissues and can be disassembled to release PTX and ICG in situ under the irradiation of a NIR laser. The released PTX, as a typical chemotherapeutic, can then induce the apoptosis of tumor cells, ensuring the tumor-targeted chemotherapy and avoiding an adverse reaction. Meanwhile, the co-released ICG, as a typical photothermic agent, endows the nanomedicine with PTT performance, thus combining chemotherapy and PTT for an enhanced and biosafe tumor nanotherapy. The self-assembly mechanism of PTX and ICG and the photo-stimulated disassembly model are explored by structural formula, hydrophilic/hydrophobic, and in situ observation. Moreover, this distinct nanomedicine, with all of its components approved by the FDA, is successfully fabricated, and its therapeutic effect against tumors is assessed both in vitro and in vivo. Comprehensively, the self-assembly and laser-mediated disassembly of PTX@ICG made it an intelligent nanomedicine for chemo/thermal therapy, which had a laser-induced therapeutic effect, a tumor-targeted drug-release, alleviated side effects, and enhanced antitumor efficiency.

## 2. Materials and Methods

Materials and reagents. Paclitaxel (PTX) and indocyanine green (ICG) were purchased from Beijing Yihe Co., Ltd. (Beijing, China) and Adamas (Shanghai, China), respectively. Paclitaxel injection was produced by Corden Pharma Latina S.P.A. (Latina, Italy). Phosphate buffered saline (PBS) was purchased from Shanghai Double-Helix Biotech Co., Ltd. (Shanghai, China). Dimethyl sulfoxide (DMSO) is a product from Shanghai Macklin Biochemical Co., Ltd. (Shanghai, China). Sodium bicarbonate (NaHCO_3_) was purchased from Sinopharm Chemical Reagent Co., Ltd. (Shanghai, China). For the agents used in the in vitro cell test, Dulbecco’s modified Eagle’s medium (DMEM), fetal bovine serum (FBS), penicillin/streptomycin and 0.25% trypsin-EDTA solution were purchased from Runcheng Biotech Co., Ltd. (Shanghai, China), and the Cell Counting Kit (CCK-8) viability assay, calcein-AM, propidium iodide (PI), and fluorescein isothiocyanate (FITC) were produced from Dojindo Laboratories (Kumamoto, Japan).

Preparation of PTX@ICG. We tried to adjust the dosage of ICG and PTX during the nanodrug preparation process to adjust the ratio of ICG and PTX in the preliminary experiment. However, the mass ratio of ICG and PTX in the final formative nanoparticles did not vary significantly in either group during the trial. Therefore, we chose the one with a ratio of ICG to PTX of about 1:2 to do the further experiment. Firstly, ICG was dissolved in an aqueous solution (1.0 mg mL^−1^) containing 0.05 mM NaHCO_3_, and 0.6 mL of this solution was transferred to a centrifuge tube. Then, under ultrasonic progressing, 0.1 mL DMSO containing PTX (10 mg mL^−1^) was added into the tube by dripping it from a disposable syringe. After that, the solution was centrifuged for 30 min under 17,000 rpm, and the precipitation was collected. The precipitation was washed by PBS to remove the solvent. Finally, the nanoparticles could be re-suspended for the next experiment or lyophilized.

Nanoparticle Characterization. We used a Zetasizer Nanoseries instrument (Nano ZS90, Malvern Instrument Ltd. Malvern, UK) to conduct the dynamic light scattering (DLS) measurements. Transmission electron microscopy (TEM) images were acquired on a JEM-2100F transmission electron microscope. Scanning electron microscopy (SEM) and scanning transmission electron microscopy (STEM) images were obtained on a field-emission S-4800 microscope (Hitachi, Japan). UV-vis-NIR absorption spectra were recorded using a UV-3600 Shimadzu UV-vis-NIR spectrometer. The quantitative analysis of components was completed by high-performance liquid chromatography (HPLC) using an Agilent 1260 Infinity II instrument. The confocal laser scanning microscopy (CLSM) experiment was conducted on an FV1000 microscope. The photothermal conversion experiment was recorded by an infrared thermal imaging instrument (FLIR A325SC camera). The laser was from an 808 nm high-power multimode pump laser produced by Shanghai Connect Fiber Optics Co., Ltd. (Shanghai, China)

ICG release of PTX@ICG. A total of 4 mL 1.0 mg mL^−1^ of nanodrugs enveloped in a dialysis bag were incubated in 46.0 mL PBS at 37 °C, and the pH of this solution was adjusted to 6.5 to mimic the faintly acidic tumor micro-environment. The concentrations of released ICG in PBS were determined by measuring the absorbance at 780 nm for ICG using a UV-vis-NIR absorption spectrum. To study the photo-stimulated ICG release, these instruments were copied and the dialysis bag was exposed to an 808 nm NIR laser at a 1.0 W cm^−2^ power density for 5 min before measurement.

Cell uptake and intracellular disassembly of PTX@ICG. To observe the cellular uptake of the PTX@ICG, the nanodrugs were dispersed in the complete DMEM (concentration was 50 μg mL^−1^) and incubated with 4T1 breast cancer cells for 8 h and 24 h, respectively. The cells were then collected and fixed on copper grids for bio-TEM observation. To study the photo-stimulated disassembly of PTX@ICG nanodrugs, these groups were copied and the Petri dishes containing cells and nanodrugs were exposed to an 808 nm NIR laser at 1.0 W cm^−2^ for 5 min after incubating for 4 h. The cells were collected and fixed for bio-TEM observation as well.

In vitro tumor cells ablation access. Murine breast cancer cells (4T1 cells, Cell Bank of Shanghai Institutes for Biological Sciences, Chinese Academy of Science) were cultured under 5% CO_2_ in Dullbecco’s modified Eagle’s medium (DMEM, GIBCO, Invitrogen, Waltham, MA, USA) and supplemented with 1% penicillin/streptomycin and 10% fetal bovine serum (FBS, Belize City, Belize) in a humidified incubator at 37 °C. Cells were plated in cell culture corning, allowed to adhere for 24 h, and then harvested by treatment with a 0.25% trypsin-EDTA solution (GIBCO, Billings, MT, USA). The cells were seeded in 96-well culture plates at a density of 1 × 10^5^ cells/well for 24 h to allow the cells to attach. Then, the culture medium above was replaced by a fresh culture medium containing PTX@ICG or other agents at different concentrations (0, 25, 50, 100 μg mL^−1^). After 24 h incubation, the standard CCK-8 viability assay (Cell Counting Kit, Dojindo Laboratories, Kumamoto, Japan) was used to evaluate the viability of the cells (n = 5). To assess the photothermal ablation effect of nanodrugs, the cells were exposed to an 808 nm laser at different power densities (0.5, 1, 1.5 W cm^−2^) for 5 min after 4 h of incubation with PTX@ICG, and then the CCK-8 assay was conducted as well.

In vivo biodistribution study. To study the biodistribution of nanodrugs in tumors and other organs, fluorescence imaging was employed to observe the biodistribution of PTX@ICG using ICG as the fluorescence probe. 4T1-tumor-bearing mice were used for FL imaging (in vivo FX PRO imaging system, Carestream Health, Singapore). Fluorescence images were acquired at different time points (1 h, 2 h, 4 h, 8 h, 12 h, 24 h, and 48 h post injection). The tumors and the major organs of the mice were harvested for ex vivo fluorescence imaging at different time points (2 h, 8 h, and 24 h post injection) to demonstrate the biodistribution of PTX@ICG.

In vivo therapeutic effect of PTX@ICG. 4T1-tumor-bearing mice were divided randomly into six groups (n = 5). The temperature rise in the tumor area was recorded by an infrared laser imaging camera. The measurement of the tumor volume was conducted by a digital caliper every 2 days for half a month, according to the formula: tumor volume = (tumor length) × (tumor width)^2^/2. The tumors and organs were dissected after the treatment period and fixed in paraformaldehyde. Then, these issues were sectioned into slices and stained with hematoxylin and eosin (H&E), TUNEL, and Ki-67 for histological analysis. After treatments, the mice were euthanized according to the standard animal protocol.

## 3. Results and Discussion

### 3.1. Preparation and Characterization of PTX@ICG

Due to the different polar functional groups of molecules, organic substances can be divided into hydrophilic, lipophilic, and amphipathic material according to their affinity with water [35]. Most chemotherapeutic drugs are hydrophobic molecules, which affects their effective availability and increases side effects. To improve the availability and relieve the side effects, a facile self-assembly method via the interaction between the hydrophobic molecules and amphipathic molecules is developed in this study. ICG, an amphipathic fluorescent dye, has been proved to be one kind of typical agent for PTT due to its desirable absorption at the near-infrared (NIR) [39,40], which has been approved by the FDA. However, free ICG has critical issues such as rapid elimination because of its small molecular size. PTX, a hydrophobic chemotherapeutic drug [41,42], is still the first-line treatment for tumor therapy. Nevertheless, the hydrophobic property of PTX causes low drug bioavailability and serious side effects in normal organs. Herein, the formation of PTX@ICG nanomedicine solved the problems encountered when they are used separately.

**Figure 1:**
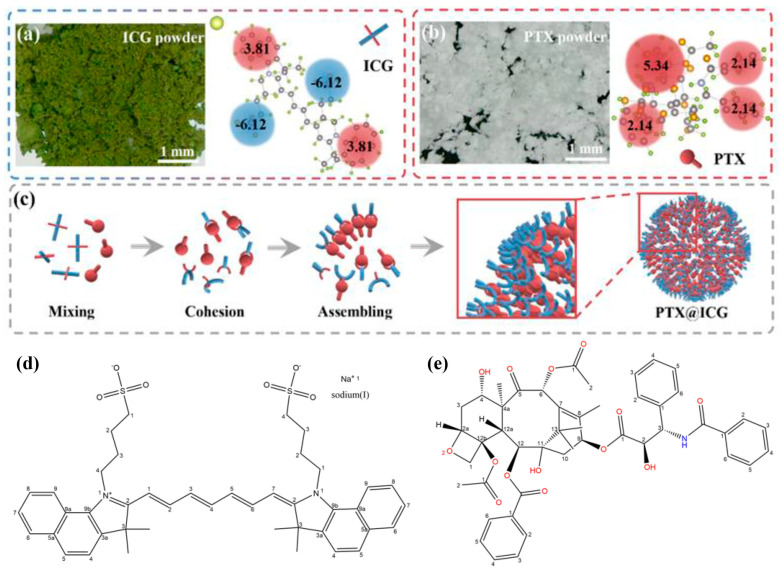
(**a**) The optical enlarged picture of ICG powder, the structural formula, and the oil-water partition coefficient of each group. (**b**) The optical enlarged picture of PTX powder, the structural formula, and the oil-water partition coefficient of each group. (**c**) Schematic diagram of the spontaneous assembly of ICG and PTX during ultrasound treatment to form a core-shell structure. The chemical structures of (**d**) ICG and (**e**) PTX.

The chemical structural formulas of ICG and PTX were drawn, and both of them were split into several groups based on their functional groups. Moreover, the oil-water partition coefficient of each group (the LogP value) was calculated to quantitatively describe the hydrophilicity and hydrophobicity [43,44]. As shown in Figure 1a,d, the LogP value of the sulfonate group in ICG exhibits the smallest negative value, which means that this group has the strongest hydrophilicity, while the LogP value of the naphthyl group is 3.81, which shows a hydrophobic property [45]. From the overall structure, ICG possesses two hydrophilic ends and two symmetrically distributed hydrophobic groups, showing a cross-like structure in space. As for PTX, the LogP values of its main groups are quite large positive values (Figure 1b,e). This explains the overall amphipathic quality of ICG, while PTX is extremely hydrophobic. Under the action of ultrasound, ICG and PTX gain energy, move continuously, and collide with each other (Figure 1c). PTX tends to combine with other PTX molecules or the hydrophobic group of ICG, which makes the hydrophilic group of ICG exposed and reduces its overall surface energy in the polar aqueous solution. Concurrently, this assembly is subjected to forces from all directions in the ultrasonic environment. Therefore, the PTX@ICG nanomedicine forms a spherical topographic core-shell structure via an ultrasound-assisted self-assembly process [46].

The optical enlarged picture (Figure S1a in Supporting Information) shows that the nanomedicine exhibits a bright green color, while ICG is a dark green powder and the color of PTX is purely white. The transmission electron microscope (TEM) image (Figure S1c) clearly exhibits the structure of as-synthesized PTX@ICG nanomedicines in that the darker PTX yolk is entirely capped by a thin ICG shell. Dynamic light scattering (DLS) measurement of PTX@ICG nanomedicines (Figure S1d) shows a sharp peak at about 105 nm. Since the nanoparticles with sizes ranging from 20 nm to 200 nm could avoid the rapid renal filtration, PTX@ICG nanomedicine was endowed with the passion accumulation property. In addition, the composition of this powder was analyzed through Fourier-transform infrared spectroscopy (FTIR) (Figure S1e). As the blue line displays, the characteristic groups in ICG, benzene and sulfonic acid salt, are distinguished. Moreover, the aromatic ester group of PTX is marked in the gray line. Unsurprisingly, the infrared characteristic peaks of these groups are all displayed in the spectrum of PTX@ICG, which reveals that it is a combination product of PTX and ICG (Figure S1e). The result of high-performance liquid chromatography (HPLC) shows that the mass ratio of ICG to PTX was about 1:2 in PTX@ICG nanoparticles.

### 3.2. The Photothermal Properties and the NIR-Activated Disassembly of PTX@ICG

To demonstrate the photothermal properties, PTX@ICG nanomedicines were exposed to 808 nm NIR laser irradiation with different concentrations at varied laser power densities (Figure S2a,b in Supporting Information). There exists a 17 °C temperature increase, which is enough to reach the 42 °C physiological limit, even though the concentration decreases to 100 μg mL^−1^ at 1.0 W cm^−2^ power density. The temperature variation of PTX@ICG nanomedicine is comparable to that of pure ICG at the same concentration, just as Figure S3a,b illustrates. After numerical calculation, the value of the extinction coefficient (ε) is quantified to be 56.09 L g^−1^ cm^−1^ (Figure S2d). As illustrated in Figure S2e (Supporting Information), the photothermal conversion efficiency (η) is calculated to be 17.9% for nanomedicines at 1.0 W cm^−2^ power density of an 808 nm NIR laser. Compared to pure ICG (24.9%), the PTX@ICG nanomedicine is similar in terms of photothermal conversion performance (Figure S2f in Supporting Information), which provides a solid foundation for effective photothermal therapy in vivo.

**Figure 2:**
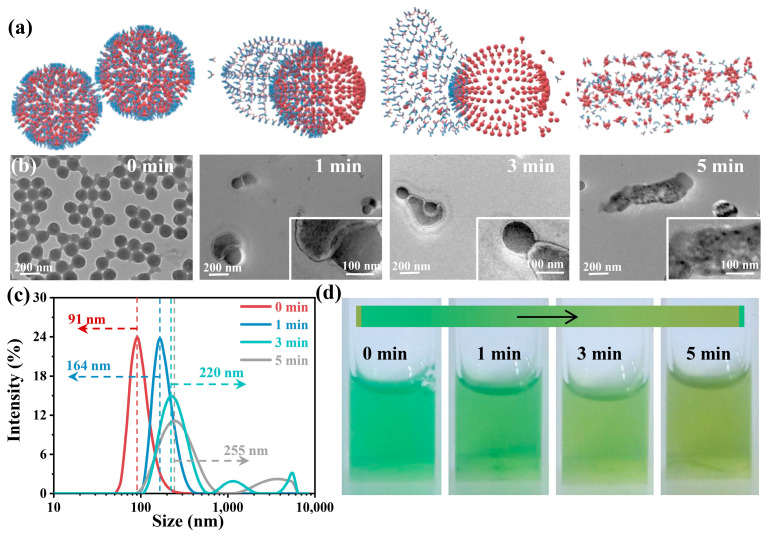
(**a**) Photo-stimulated disassembly process of PTX@ICG nanomedicines. (**b**) TEM images showing the morphology changes of nanomedicines along with the extension of irradiation time. (**c**) The size transformation during the laser irradiation. (**d**) The color changes of aqueous suspension during the period of laser irradiation.

The controllable disassembly of PTX@ICG with NIR activation as a switch is detected in Figure 2. The PTX@ICG under laser irradiation is in a higher energy state, which means the stable PTX@ICG can change structure due to the participation of exogenous energy input. As a result, ICG molecules take off from the composite and make the core-shell structure change to a hat-like structure. With the continuous light irradiation, more ICG molecules break away and more PTX molecules are exposed (Figure 2a,b). The cluster of PTX cannot remain a spherical morphology in aqueous solution, and the PTX tends to agglomerate, which can be verified by the TEM images in Figure 2b. The DLS analysis also externalizes that the size of the nanomedicine increases gradually from 91 nm to 255 nm during the irradiation period and at last generates precipitation (Figure 2c). Moreover, under the motivation of the NIR laser, the color of the solution changes from green to canary yellow, as shown in Figure 2d, which indicates the altered structure for PTX@ICG. Furthermore, the amount of released ICG from nanomedicines was measured by UV-vis-NIR spectra (Figure S4): the releasing ratio of ICG reached 60% in two days (pH = 6.5) and the release rate of ICG increased, almost reaching 80% after exposure to the NIR laser. These results demonstrate that PTX@ICG could quickly disassemble at the tumor area under NIR laser stimuli, and photothermal-motivated chemotherapy could be conducted. 

To achieve the local tumor chemotherapy without side effects, no disintegration at non-targeted sites is necessary for the nanomaterials in a physiological environment. The stability and the zeta potentials of PTX@ICG in several simulated body fluids, such as PBS, normal saline (NS), and a mixed solution containing 10% fetal bovine serum (FBS), were tested. As shown in Figure S5, the zeta potentials of PTX@ICG nanoparticles were tested to be about −18 mV, −25 mV, and −15 mV, respectively, in normal saline, PBS, and 10% FBS solutions. Furthermore, the TEM images of PTX@ICG (Figure S6a–c) illustrate that it could keep the original structure without disintegration for 5 days. Further, all samples can remain at high dispersity during the body circulation period, as the DLS curves display (Figure S6d–f). Additionally, the exact same peak shape of absorbance from 0 days to 5 days in vis-NIR absorption spectra (Figure S6g–i) expresses that the coating state of PTX@ICG is maintained after storage for a long time, and PTX is scarcely released from PTX@ICG in an unexpected area. 

### 3.3. In Vitro Chemo-Photothermal Therapy of PTX@ICG

**Figure 3:**
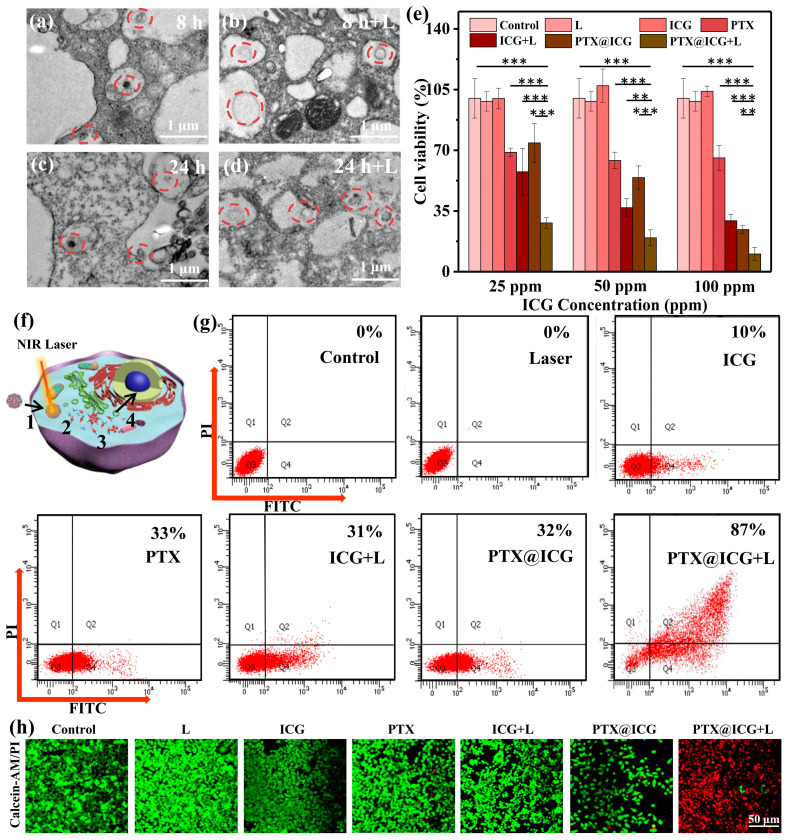
Bio-TEM of 4T1 tumor cells and PTX@ICG (50 ppm in ICG concentration) with different treatments: (**a**) 8 h incubation, (**b**) 8 h incubation and laser irradiation (1 W cm^−2^), (**c**) 24 h incubation, (**d**) 24 h incubation and laser irradiation. (**e**) Viabilities of 4T1 tumor cells tested by standard CCK-8 assay. Cells are incubated with laser irradiation, ICG, PTX, ICG and laser, PTX@ICG, PTX@ICG and laser for 24 h at different concentrations (25 ppm, 50 ppm, 100 ppm in ICG concentration), respectively (*n* = 5, mean ± SD; **, *p* < 0.05; ***, *p* < 0.001). (**f**) Schematic diagram of the treatment principle at the cell level. Tumor cell endocytosis of PTX@ICG (1); Photothermal therapy by ICG (2); Photothermal-promoted disassembly process (3); PTX-induced chemotherapy by blocking the cell proliferation cycle (4). (**g**) Flow cytometry characterization of cellular apoptosis and the sum of early and late apoptosis. Cells are incubated with laser irradiation, ICG, PTX, ICG and laser, PTX@ICG, PTX@ICG and laser for 24 h at 100 ppm in ICG concentration, respectively. (**h**) CLSM imaging of cell apoptosis after various treatments (laser irradiation, ICG, PTX, ICG and laser, PTX@ICG, PTX@ICG and laser for 24 h at 100 ppm in ICG concentration). Live 4T1 cells are stained with calcein-AM (green) and dead cells are stained with PI (red).

In order to realize the final antitumor application, the in vitro endocytosis and cell ablation were carried out. The breast cancer cells (4T1 cells) were incubated with 50 ppm PTX@ICG for different durations (8 h/24 h) and different treatments (with/without NIR laser stimulating after 4 h post incubation). For the sample incubated for 8 h without laser treatment in Figure 3a, there are many black dots in the cell’s vesicles, marked by red circles, which are nanomedicines swallowed by the cell’s endocytosis. After incubation for 24 h (Figure 3c), the nanomedicines disassemble partly or entirely in tumor cells. As for the samples with laser radiation (Figure 3b,d), it should be noticed that the PTX@ICG is broken and spreads in a wider range compared to those without laser irradiation. This result means that the disassembly progress in tumor cells is slightly spontaneous and can be accelerated substantially by NIR stimulation.

After the confirmation of endocytosis performance, the in vitro cytotoxicity tests of PTX@ICG were also carried out on 4T1 tumor cells using a typical CCK-8 assay (Figure 3e). The same number of cells were incubated with PTX, ICG, and PTX@ICG for 24 h at different concentrations (25 ppm, 50 ppm, 100 ppm in ICG concentration), respectively. Moreover, the photothermal ablation performance was achieved by NIR laser stimulation after incubation for 4 h. Synthetically, a slightly low power density of 1.0 W cm^−2^ was chosen to ensure biosecurity. As shown in Figure 3e, in the sample treated only by laser, the cell viability remains at 98%, confirming that a low-energy density laser does not damage the cells. For the group treated with ICG and laser, the cell viabilities decreased to 57%, 36%, and 29%, respectively, at the same concentrations as above. For the PTX@ICG group, the cell viabilities are 74%, 54%, and 24% at 25 ppm, 50 ppm, and 100 ppm, respectively, and the cell viabilities decrease sharply to 29%, 19%, and 10% for tumor cells treated with PTX@ICG and NIR laser radiation. These phenomena indicate that ICG or laser only cannot decrease the survival cell rate and can manage it only under the condition of a combination; PTX can effectively reduce the percent of survival tumor cells to 60%, and PTX@ICG with the laser has a better effect than the therapeutic methods of PTX and ICG with the laser, confirming the effective synergistic anti-tumor effect of PTX@ICG-induced chemo-photothermal therapy.

The schematic diagram of the treatment principle at the cell level is displayed in Figure 3f. PTX@ICG are endocytosed into the cytoplasm by tumor cells due to an appropriate size effect. Under the stimulation of NIR, ICG converts light energy into heat energy and exerts a photothermal therapy effect. At the same time, PTX@ICG gradually disintegrates to release the PTX, which can further enter the nucleus of tumor cells to block the cell proliferation cycle. The flow cytometry analysis, as exhibited in Figure 3g, shows the same trend and results with the cell viability experiment, which demonstrates the decent tumor ablation capacity of PTX@ICG at the safe laser-power density. To demonstrate the therapeutic effect intuitively, confocal laser scanning microscopy (CLSM) (Figure 3h) was used to observe the viability of the tumor cells. The number of live cells in the PTX group and the ICG with laser group decreased, but the red signals are weak. That is because PI can only stain necrotic or late apoptotic cells but not early apoptotic cells. The other reason is that the low adhesion force of dead cells causes the wastage of red signals in the visual field. Furthermore, the cells incubated with PTX@ICG have a weaker green signal and a stronger red signal than PTX and ICG with laser, which means that PTX@ICG features a favorable anti-tumor cell effect via improving the utilization of PTX. Matching the above cell-viability experiment and flow-cytometry analysis, PTX@ICG with laser irradiation shows hardly any green signal, suggesting that nearly all tumor cells are killed by the coordination of PTT and laser-triggered disassembly induced chemotherapy.

### 3.4. The Bio-Distribution of PTX@ICG

As the superiority of PTX@ICG was verified in vitro, the pharmacokinetic parameters of PTX@ICG in vivo were studied to confirm the potential application in the clinic. The fluorescent tracer ability of ICG was applied to show the distribution of nanomedicines in vivo, as it has already been used as a clinical fluorescent dye for the diagnosis of liver disease [47]. The distribution of PTX@ICG (Figure S7a) was recorded by the whole-body fluorescent imaging system in time gradient. As time goes by, the fluorescent intensity in the tumor area gradually increases at first and reaches a maximum value at 8 h post injection. When the fluorescent intensities in other regions of the body quickly decrease, that of the tumor area still remains a higher value during the observation period, indicating the good enrichment and retention effect of PTX@ICG in tumor tissues. The fluorescent images of isolated organs (Figure S7b) at different times also clearly exhibit a similar distribution in organs. This phenomenon indicates that PTX@ICG can effectively enrich and stay in the tumor region for a long time, which guarantees the following treatment. These results co-confirm that the PTX@ICG not only has good dispersibility and biocompatibility in simulated body environments but also possesses a longer blood circulation period and passive accumulation in the tumor area by the EPR effect, which makes the combination therapy capable.

### 3.5. In Vivo Chemo-Photothermal Therapy of PTX@ICG

**Figure 4:**
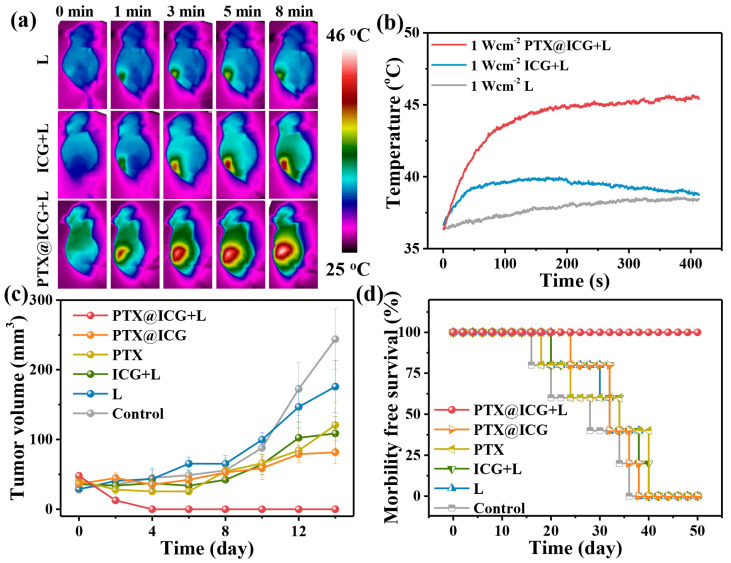
(**a**) Infrared imaging of 4T1-tumor-bearing mice irradiated (1.0 W cm^−2^) at the tumor site. Mice are treated with NS (intravenously injected, the same below) and laser, free ICG and laser, PTX@ICG and laser, respectively. (**b**) The temperature changes at the tumor area treated with laser (1.0 W cm^−2^), free ICG and laser, PTX@ICG and laser, respectively. (**c**) tumor volume changing curves of 4T1-tumor-bearing mice treated with NS, laser irradiation, pure PTX (6.67 mg kg^−1^), ICG (3.33 mg kg^−1^) and laser irradiation, PTX@ICG (6.67 mg kg^−1^ in PTX concentration), and PTX@ICG (6.67 mg kg^−1^ in PTX concentration) combining laser, respectively. (*n* = 5, mean ± SD). (**d**) Survival curves of mice with treatments mentioned above.

After elucidating the tumor-targeting properties of PTX@ICG, the therapeutic effect in vivo was further researched. The tumor-bearing mice were randomly divided into six groups and injected intravenously with PTX@ICG, ICG, or PTX at the same ICG concentration of 3.33 mg kg^−1^ and a PTX concentration of 6.67 mg kg^−1^, respectively. Then, the tumor site was laser irradiated with an 808 nm NIR laser for 10 min at 8 h post injection. The temperature of the tumor treated with PTX@ICG (Figure 4a,b) rose to 46 °C under NIR laser irradiation, which is far more than the cell’s physiological limit, while the ICG with the laser did not heat up efficiently. This is because the nanosize of PTX@ICG ensures its circulation in vivo and tumor enrichment. 

As shown in Figure 4c, the photothermal effect of ICG only and chemotherapy of PTX only both have a slight inhibitory effect on tumor growth, while the anti-tumor effect of PTX@ICG is slightly better than the above two monotherapies. This is because the formed nanoparticles can accumulate in the tumor area effectively via the EPR effect and enhance the therapeutic effect. In contrast, the PTX@ICG combination therapy can completely ablate the tumor and significantly prevent its recurrence. The combination index of the PTX@ICG-mediated synergistic photothermal-chemotherapy is calculated to be 1.24, according to King’s formula [47]. The dissected tumor after different treatments (Figure S8) clearly shows the tumor growth inhibition of different groups. 

At the end of the two-week treatment period, the mice continued to be observed until 50 days (Figure 4d), and the mice in all the other groups gradually died; only the mice that were treated with the PTX@ICG combined with laser still maintained vitality. Moreover, the tissue slices (Figure S9 in Supporting Information) of the heart, liver, spleen, lung, and kidney isolated from the PTX@ICG with laser group after the therapeutic progress show no obvious difference compared to the control group. Furthermore, the hematological indexes (Figure S10 in Supporting Information) of Kunming mice treated with 20 mg kg^−1^ PTX@ICG had no significant changes compared to the blank control group. All these pieces of evidence confirm the biosecurity of PTX@ICG.

## 4. Conclusions

In conclusion, a novel kind of carrier-free nanomedicine composed of only two clinical drugs was successfully prepared through a simple ultrasound-assisted assembly method in this work. Two components, PTX and ICG, joined together via the interaction of hydrophilicity and hydrophobicity, which was verified by in situ observation. The nanomedicine possesses good dispersibility, and there is no need for further surface decoration. The PTX’s chemotherapy and the ICG’s PTT effect are combined in this nanomedicine, creating excellent performance for anti-cancer both in vitro and in vivo. Furthermore, according to the efficient accumulation in the tumor area and the laser-stimulated disassembly phenomenon found in the photothermal test, the nanomedicine can work only in the local tumor environment, which further ensures its decent biosecurity and high efficiency. Thus, this kind of nanomedicine, which is synthesized by facile self-assembly with excellent therapeutic effects and great biocompatibility, provides a distinct paradigm to achieve high tumor-treatment efficacy and deserves to be further explored.

## Data Availability

The data that support the findings of this study are available from the corresponding authors upon reasonable request.

## Supplementary Materials

The following supporting information can be downloaded at: https://www.mdpi.com/article/10.3390/nano13182499/s1, Figure S1: (a) Optical magnification picture of PTX@ICG nanomedicines. (b) SEM image of PTX@ICG. (c) TEM image and enlargement of the core-shell part. (d) Size dispersion of PTX@ICG (inset shows the digital photo of PTX@ICG dispersed in PBS and the Tyndall effect). (e) Infrared transmittance spectrum of PTX, ICG, PTX@ICG nanomedicines and peaks of several characteristic groups. (f) UV-vis-NIR Absorption spectra of ICG and PTX@ICG; Figure S2: Temperature elevation of PTX@ICG nanomedicine at (a) 1.0 W cm^−2^ and (b) 1.5 W cm^−2^ power density. (c) Vis-NIR absorbance properties of aqueous solutions containing PTX@ICG in different concentrations. (d) The extinction coefficient of PTX@ICG nanomedicine at 808 nm. (e) The photothermal conversion efficiency of PTX@ICG nanomedicine at 1 W cm^−2^ power density. (f) The photothermal conversion efficiency of pure ICG at 1 W cm^−2^ power density; Figure S3: Temperature rise of pure ICG at (a) 1.5 W cm^−2^ power density, (b) 1 W cm^−2^ power density; Figure S4: The amount of released ICG from nanodrugs within two days with and without laser irradiation; Figure S5: Zeta potentials of PTX@ICG dispersed in NS, PBS and 10% FBS; Figure S6: TEM images of PTX@ICG dispersed in (a) NS, (b) PBS, (c) 10% FBS after 5 days. (d–f) Size variation of PTX@ICG nanomedicines in different solutions in 5 days. (g–i) UV-vis-NIR absorption spectra of nanomedicines dispersed in different solutions; Figure S7: (a) Whole-body fluorescent imaging of PTX@ICG (10 mg kg^−1^ in PTX concentration) in 4T1 tumor-bearing mice at different time (1 h, 2 h, 4 h, 8 h, 12 h, 24 h, 48 h) post-injection. (b) The fluorescent imaging of isolated organs (heart, liver, spleen, lung, kidney) and tumor at 2 h, 8 h, 24 h post-injection; Figure S8: Digital photograph of tumor dissected after 14 days treatment; Figure S9: H&E immunofluorescence staining of organs (heart, liver, spleen, lung, kidney) treated with normal saline, PTX@ICG and laser to compare the difference and prove the biological safety of PTX@ICG. Organs are isolated after a two-week treatment period; Figure S10: (a) During 30 days feeding, the in vivo body weight curve of Kunming mice treated with PBS, free PTX, 10 mg/kg Nanodrugs and 20 mg/kg Nanodrugs respectively. (b–l) Hematological index of Kunming mice after 30 days observation. (see Ref. [48]).
