# Supplementary material for: Engineering Self-Assembled Nanomedicines Composed of Clinically Approved Medicines for Enhanced Tumor Nanotherapy
Source: Nanomaterials (Basel). 2023 Sep 5;13(18):2499. doi: 10.3390/nano13182499 (PMC10534536; doi:10.3390/nano13182499)
Supplement: Supplementary file 1 [file nanomaterials-13-02499-s001.zip › nanomaterials-2521892-supplementary.pdf]

Supporting information

Engineering self-assembled nanomedicines composed of clinically approved medicines for enhanced tumor nanotherapy

Quzi Jiang[a,b], Luodan Yu[c], and Yu Chen[a,c,]*

[a]*Shanghai Institute of Ceramics, Chinese Academy of Sciences, Shanghai 200050, P. R. China*

[b]*University of Chinese Academy of Sciences, Beijing, 100049, P. R. China*

[c]*School of Life Sciences, Shanghai University, Shanghai 200444, P. R. China*

Corresponding author: chenyuedu@shu.edu.cn

Method

Photothermal effect of PTX@ICG.

Photothermal performance was obtained by exposing a 96-well plate containing a dispersion of PTX@ICG and recorded by an infrared thermal imaging camera.

To calculate the extinction coefficient of nanodrugs, the Lambert-Beer law was employed.

$A(\lambda) = \varepsilon LC$

A is the absorbance at the wavelength of λ, L is the path length (in our experiment is 1.0 cm) and C (in g L^{-1}) is the concentration of the nanodrugs. The extinction coefficient (ε) is calculated by plotting the slope of each linear fit against wavelength.

To calculate the photothermal conversion efficiency (η), the method reported by Roper et al. was adopted [1].

$\eta = [hS(T_m - T_s) - Q_{Dis}] \div I(1 - 10^{(-A\_\lambda)})$

where A_λ, L, and C are the absorbance at the specific wavelength (λ), the path length (1.0 cm), and the concentration of the nanodrugs (in g L^{-1}). The h, S, T_m, T_s, Q_{Dis} and I refer to heat-transfer coefficient, the surface area of the container, the equilibrium temperature of system surface, the surrounding temperature, the heat dissipated from the photoabsorption of the quartz cuvette sample cell itself, and the incident energy of the NIR laser (mW), respectively.

Calculation of the combination index of ICG@PTX-induced photothermal-chemotherapy.

The combination index was calculated according to the King's formula:

$Q = E_{a+b}/(E_a + E_b - E_a \times E_b)$

Where E_{a+b}, E_a and E_b are the antitumor efficiency of photothermal-chemotherapy of ICG@PTX, photothermal therapy of ICG and chemotherapy of PTX, respectively.

Reference

[1] Roper, D.K.; Ahn, W.; Hoepfner, M. Microscale heat transfer transduced by surface plasmon resonant gold nanoparticles. *J. Phys. Chem. C* **2007**, *111*, 3636–3641.

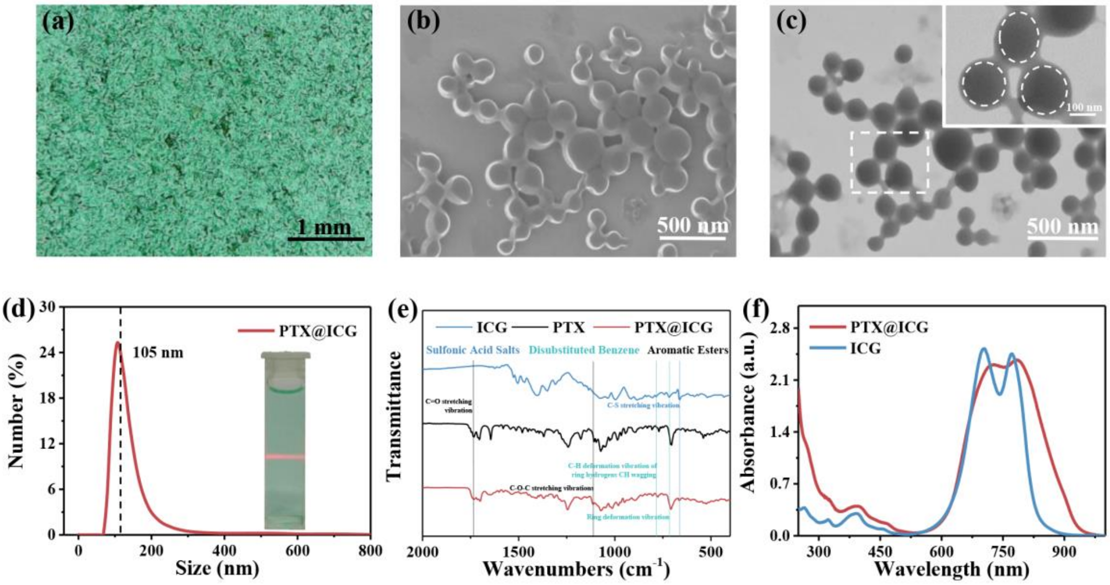

Figure S1. (a) Optical magnification picture of PTX@ICG nanomedicines. (b) SEM image of PTX@ICG. (c) TEM image and enlargement of the core-shell part. (d) Size dispersion of PTX@ICG (inset shows the digital photo of PTX@ICG dispersed in PBS and the Tyndall effect). (e) Infrared transmittance spectrum of PTX, ICG, PTX@ICG nanomedicines and peaks of several characteristic groups. (f) UV-vis-NIR Absorption spectra of ICG and PTX@ICG.

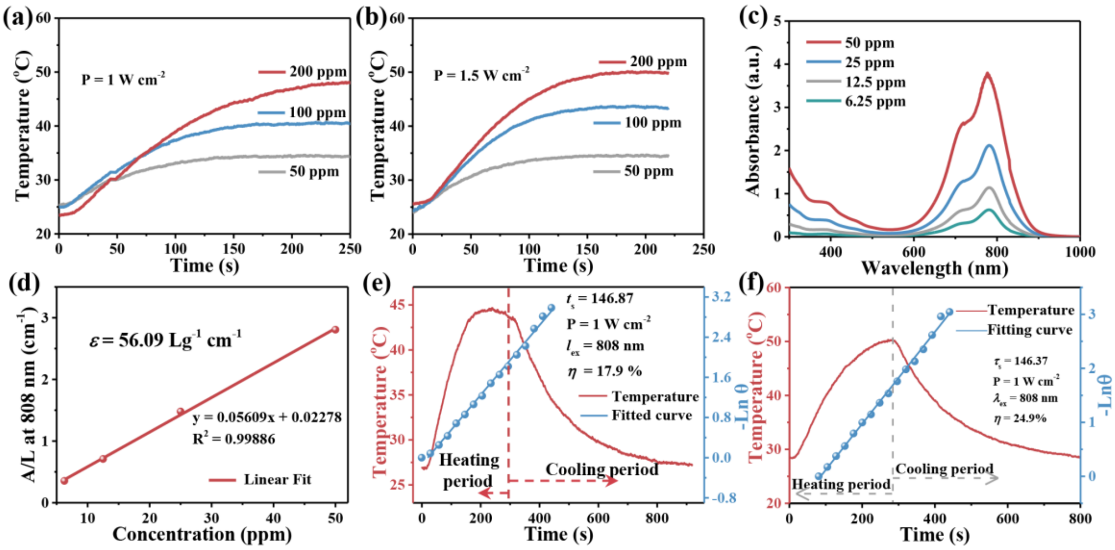

Figure S2. Temperature elevation of PTX@ICG nanomedicine at (a) 1.0 W cm^{-2} and (b) 1.5 W cm^{-2} power density. (c) Vis-NIR absorbance properties of aqueous solutions containing PTX@ICG in different concentrations. (d) The extinction coefficient of PTX@ICG nanomedicine at 808 nm. (e) The photothermal conversion efficiency of PTX@ICG nanomedicine at 1 W cm^{-2} power density. (f) The photothermal conversion efficiency of pure ICG at 1 W cm^{-2} power density.

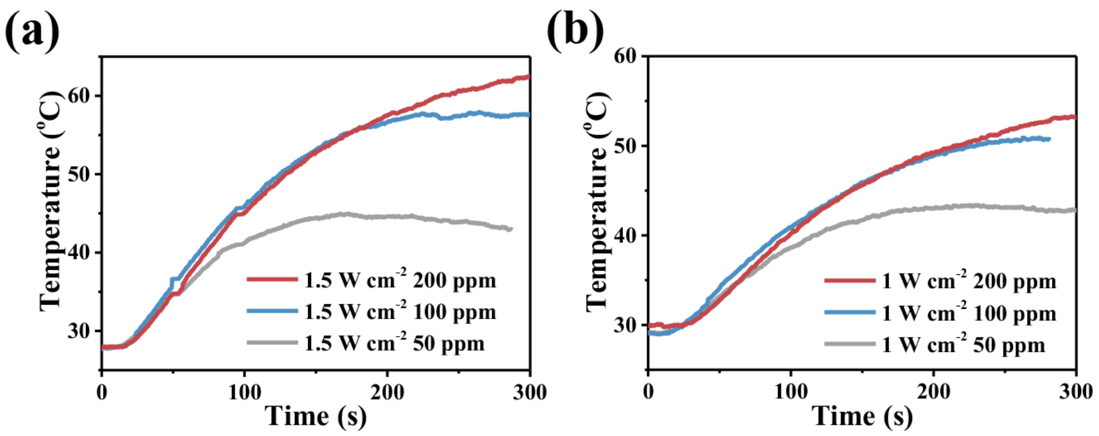

Figure S3. Temperature rise of pure ICG at (a) 1.5 W cm^{-2} power density, (b) 1 W cm^{-2} power density.

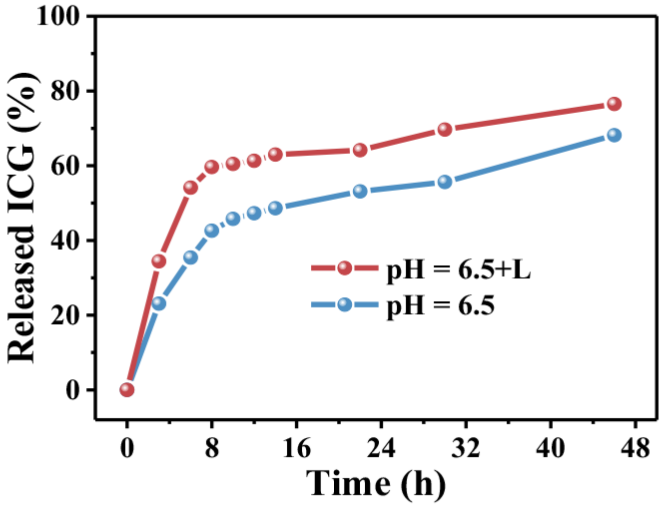

Figure S4. The amount of released ICG from nanodrugs within two days with and without laser irradiation.

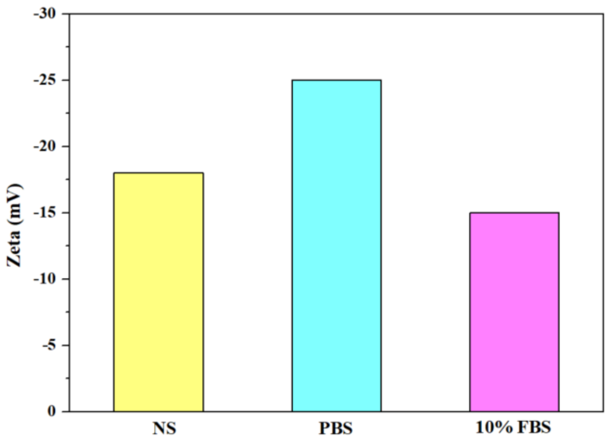

Figure S5. Zeta potentials of PTX@ICG dispersed in NS, PBS and 10% FBS.

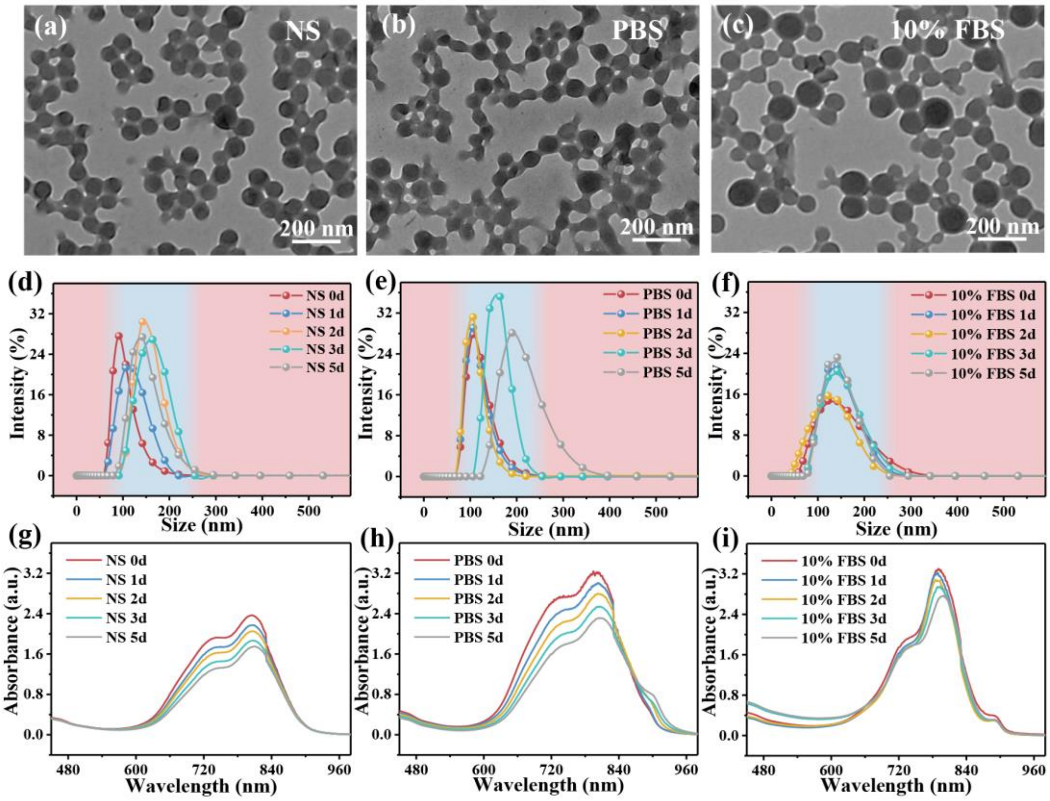

Figure S6. TEM images of PTX@ICG dispersed in (a) NS, (b) PBS, (c) 10% FBS after 5 days. (d-f) Size variation of PTX@ICG nanomedicines in different solutions in 5 days. (g-i) UV-vis-NIR absorption spectra of nanomedicines dispersed in different solutions.

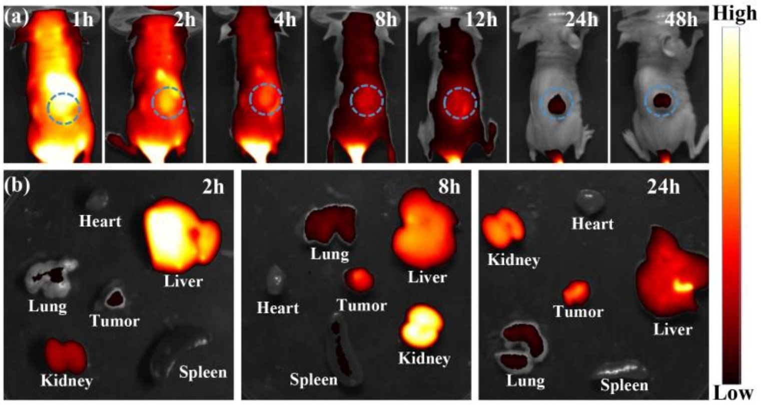

Figure S7. (a) Whole-body fluorescent imaging of PTX@ICG (10 mg kg^{-1} in PTX concentration) in 4T1 tumor-bearing mice at different time (1h, 2h, 4h, 8h, 12h, 24h, 48h) post-injection. (b) The fluorescent imaging of isolated organs (heart, liver, spleen, lung, kidney) and tumor at 2 h, 8 h, 24 h post-injection.

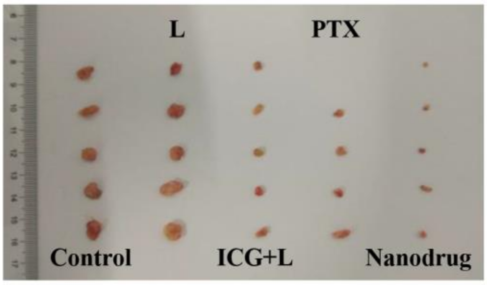

Figure S8. Digital photograph of tumor dissected after 14 days treatment.

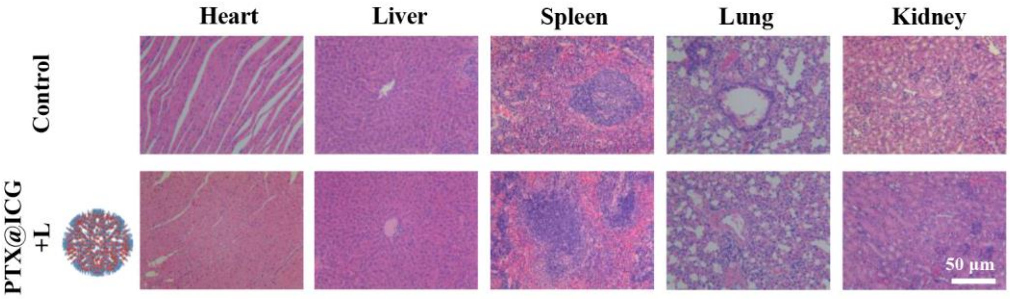

Figure S9. H&E immunofluorescence staining of organs (heart, liver, spleen, lung, kidney) treated with normal saline, PTX@ICG and laser to compare the difference and prove the biological safety of PTX@ICG. Organs are isolated after a two-week treatment period.

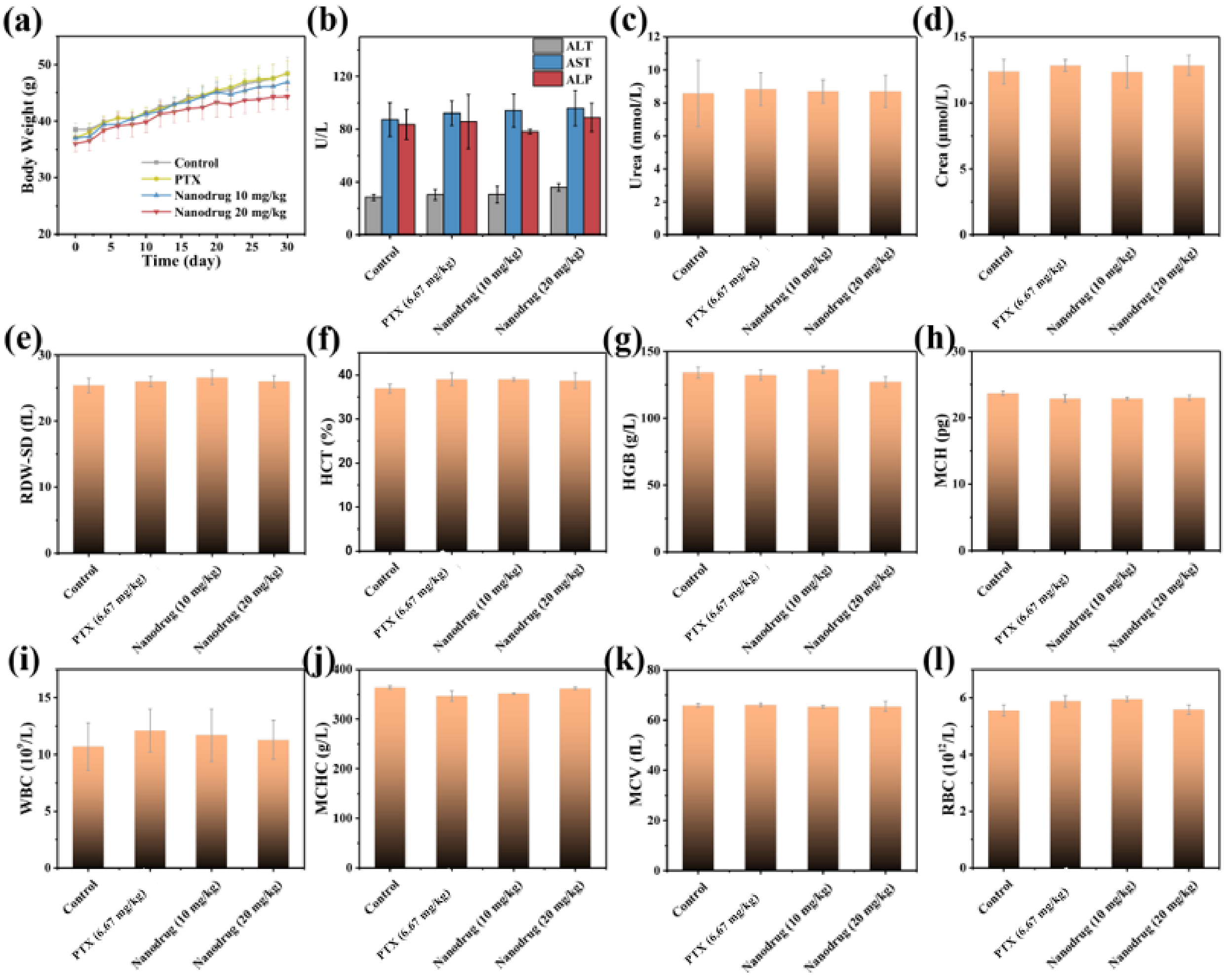

Figure S10. (a) During 30 days feeding, the in vivo body weight curve of Kunming mice treated with PBS, free PTX, 10 mg/kg Nanodrugs and 20 mg/kg Nanodrugs respectively. (b)-(l) Hematological index of Kunming mice after 30 days observation.
